# Successful Conversion from Conventional Potassium Binder to Sodium Zirconium Cyclosilicate in a Patient with Refractory Constipation

**DOI:** 10.3390/medicina58050635

**Published:** 2022-05-03

**Authors:** Teruhiko Imamura, Koichiro Kinugawa

**Affiliations:** The Second Department of Internal Medicine, University of Toyama, 2630 Sugitani Toyama, Toyama 930-0194, Japan; kinugawa-tky@umin.ac.jp

**Keywords:** hyperkalemia, chronic kidney disease, heart failure

## Abstract

Potassium binders are essential tools to treat hyperkalemia, particularly in patients with heart failure and chronic kidney disease. One of the drug-related complications is constipation, which further worsens heart failure by increasing afterload and decreases patients’ quality of life. We encountered an 82-year-old man with heart failure, chronic kidney disease, and hyperkalemia. A conventional potassium binder, calcium polystyrene sulfonate, ameliorated his hyperkalemia, whereas he became aware of refractory constipation. A conversion to a newly-introduced specific potassium binder, sodium zirconium cyclosilicate, improved persistent constipation, maintaining serum potassium levels within a normal range. Sodium zirconium cyclosilicate might be a preferable potassium binder to treat hyperkalemia, particularly in patients with heart failure and chronic kidney disease and those suffering from constipation.

## 1. Introduction

Management of hyperkalemia using conventional potassium binders is of great importance to improve mortality and morbidity, particularly for those with heart failure and chronic kidney disease and those receiving renin-angiotensin system inhibitors [1]. Constipation is one of many major drug-related complications, which worsens heart failure by increasing afterload and decreases patients’ quality of life [2]. 

Recently, sodium zirconium cyclosilicate (SZC), a zirconium silicate compound that exchanges sodium and hydrogen for potassium and ammonium ions in the gastrointestinal tract, was reimbursed to lower serum potassium levels with robust evidence [3]. Theoretically, given its non-polymer nature, SZC might be less associated with drug-related constipation compared with the conventional potassium binders [4]. However, there is a scarcity of reports that displayed the advantage of SZC in reducing the incidence and/or degree of drug-related constipation over the conventional ones. 

We encountered a patient with heart failure and preserved ejection fraction, chronic kidney disease, and hyperkalemia. Potassium binder-related refractory constipation was ameliorated by the conversion to SZC, maintaining his serum potassium level within the normal range. 

## 2. Case Report

### 2.1. Medical History

An 82-year-old man with heart failure and preserved ejection fraction due to hypertensive heart disease was referred to our outpatient clinic for a follow-up. He received coronary artery bypass grafting 28 years ago. His comorbidities were chronic kidney disease and dyslipidemia. He had received aspirin 100 mg, famotizine 10 mg, atorvastatin 20 mg, amlozipine 5 mg, and azilsartan 40 mg. 

### 2.2. On Referral

His height was 160 cm and his weight was 60 kg. Blood pressure was 143/61 mmHg and pulse rate was 70 bpm. His New York Heart Association functional class was II. Chest X-ray displayed no cardiomegaly nor pulmonary congestion. Electrocardiogram revealed 76 bpm of heart rate with sinus rhythm. Transthoracic echocardiography depicted 49 mm of left ventricular end-diastolic diameter and 69% of left ventricular ejection fraction with mild aortic stenosis. E/e’ ratio was 13.5. 

**Figure 1:**
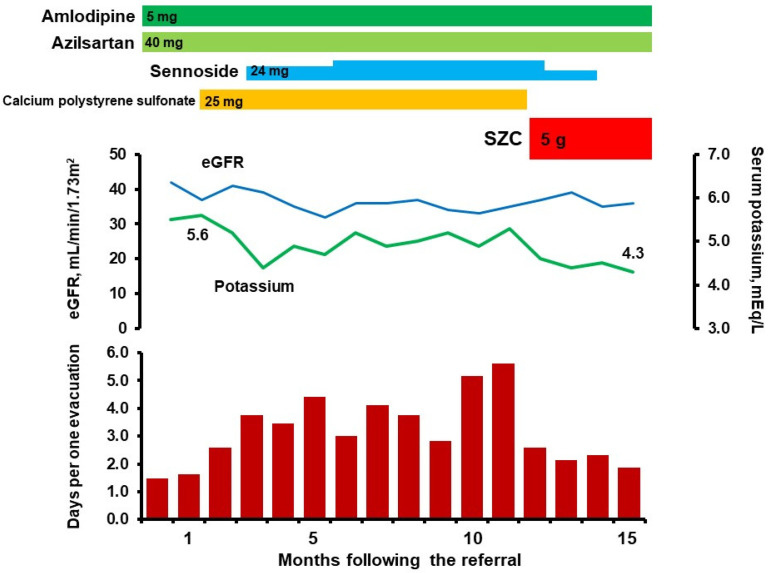
Clinical course following the referral. Hyperkalemia was treated by calcium polystyrene sulfonate and constipation refractory to sennoside developed gradually. Following the conversion to SZC, constipation improved considerably with a maintained serum potassium level around 4.5 mEq/L. eGFR, estimated glomerular filtration ratio.

Plasma B-type natriuretic peptide level was 134 pg/mL. Serum potassium level was 5.5 mEq/L and estimated glomerular filtration ratio was 42 mL/min/1.73 m^2^ (stage G3b, Figure 1). His serum potassium level trended around 5.5 mEq/L before the referral. We initiated calcium polystyrene sulfonate 25 mg for hyperkalemia and asked him for mild dietary potassium restriction according to the guidelines [5]. He had never complained of constipation before the referral (averaged days per one evacuation had been 1.5).

### 2.3. Follow-Up

Two months following the initiation of calcium polystyrene sulfonate, his serum potassium level decreased to 4.4 mEq/L, whereas he reported worsening and symptomatic constipation with abdominal distension. Averaged days per one evacuation increased up to 3.8 with a constipation scale of 9/16. We initiated sennoside 24 mg, which was further up-titrated later for his refractory constipation. 

At 11 months following the referral, averaged days per one evacuation increased up to 5.6 with a constipation scale of 13/16. Serum potassium level was controlled at around 5.0 mEq/L. We decided to convert calcium polystyrene sulfonate to SZC 5 mg. 

Following the conversion, his constipation improved considerably with averaged days per one evacuation down to around 2.0 and a constipation scale decreased down to 4/16. Sennoside was weaned off. Serum potassium levels were controlled at around 4.5 mEq/L and plasma B-type natriuretic peptide level was 67 pg/mL at 15 months following the referral. 

## 3. Discussion

### 3.1. Potassium Binders to Treat Hyperkalemia

Several conventional potassium binders including calcium polystyrene sulfonate, which we initially prescribed, have been available to treat hyperkalemia [1], but their safety and efficacy have not been demonstrated in large-scale trials. 

The HARMONIZE phase III study demonstrated that 30-day 5 or 10 g SZC therapy ameliorated hyperkalemia with acceptable safety [6]. The trial included only 19% of heart failure patients. Our team also demonstrated that SZC could assist to up-titrate renin-angiotensin-aldosterone system inhibitors by improving hyperkalemia in patients with heart failure [7]. 

Our patient had hyperkalemia dominantly due to the existence of chronic kidney disease and the use of renin-angiotensin system inhibitor. We converted calcium polystyrene sulfonate to SZC, which further stabilized the serum potassium levels from around 5.0 mEq/L to around 4.5 mEq/L. We selected a maintenance dose of 5 g/day, instead of the loading dose of 30 g/day, as an initial dose to avoid hypokalemia by the aggressive loading of SZC [8].

### 3.2. Potassium Binders and Constipation

Constipation is often encountered in patients with heart failure and chronic kidney disease [9]. Cardiac cachexia and visceral congestion decrease bowel motility. The use of diuretics causes hard stool. Uremia disrupts the stability of gut bacteria. Potassium restriction to treat concomitant hyperkalemia decreases the intake of dietary fiber. 

He became aware of refractory constipation soon after the initiation of calcium polystyrene sulfonate and restriction of potassium intake to treat his hyperkalemia. Constipation is one of the major complications of these conventional potassium binders [2]. Detailed mechanisms for this condition are unknown, but these polymers would expand and stay longer in the bowel when deionized water coexists [4].

Constipation should be appropriately managed particularly for those with heart failure (since the effort against constipation would increase afterload and worsen heart failure) [9].

In the in vivo study, the volume of SZC rather decreased when deionized water was added, probably because SZC is non-polymer, which is one of the unique features of SZC compared with other conventional potassium binders [4]. Furthermore, the dose of SZC that we prescribed was only 5 g per day as compared to 25 g of calcium polystyrene sulfonate. In the HARMONIZE phase III trial, the incidence of constipation was below 10% [6].

These were the reasons why we converted conventional potassium binder to SZC. SZC might be preferred to conventional potassium binders particularly in patients with ongoing or potential constipation, together with heart failure and chronic kidney disease. We should emphasize that SZC was not administered to treat constipation. SZC was administered to treat hyperkalemia. SZC might have an advantage in less causing drug-related constipation compared to the conventional potassium binders.

In conclusion, SZC might be preferred to conventional potassium binders to treat hyperkalemia, particularly in patients with heart failure, chronic kidney disease, and ongoing/potential constipation. Conversion from conventional potassium binders to SZC might be recommended when drug-related constipation is suspected.

## Data Availability

Data are available from the corresponding authors upon reasonable requests.

## References

[1] Sarwar C.M., Papadimitriou L., Pitt B., Pina I., Zannad F., Anker S.D., Gheorghiade M., Butler J. (2016). Hyperkalemia in Heart Failure. J. Am. Coll. Cardiol..

[2] Harel Z., Harel S., Shah P.S., Wald R., Perl J., Bell C.M. (2013). Gastrointestinal adverse events with sodium polystyrene sulfonate (Kayexalate) use: A systematic review. Am. J. Med..

[3] Hoy S.M. (2018). Sodium Zirconium Cyclosilicate: A Review in Hyperkalaemia. Drugs.

[4] Stavros F., Yang A., Leon A., Nuttall M., Rasmussen H.S. (2014). Characterization of structure and function of ZS-9, a K+ selective ion trap. PLoS ONE.

[5] McDonagh T.A., Metra M., Adamo M., Gardner R.S., Baumbach A., Bohm M., Burri H., Butler J., Celutkiene J., Chioncel O. (2021). 2021 ESC Guidelines for the diagnosis and treatment of acute and chronic heart failure. Eur. Heart J..

[6] Zannad F., Hsu B.G., Maeda Y., Shin S.K., Vishneva E.M., Rensfeldt M., Eklund S., Zhao J. (2020). Efficacy and safety of sodium zirconium cyclosilicate for hyperkalaemia: The randomized, placebo-controlled HARMONIZE-Global study. ESC Heart Fail..

[7] Imamura T., Oshima A., Narang N., Kinugawa K. (2021). Clinical Implications of Sodium Zirconium Cyclosilicate Therapy in Patients with Systolic Heart Failure and Hyperkalemia. J. Clin. Med..

[8] Oshima A., Imamura T., Narang N., Kinugawa K. (2021). Management of hyperkalemia in chronic heart failure using sodium zirconium cyclosilicate. Clin. Cardiol..

[9] Ishiyama Y., Hoshide S., Mizuno H., Kario K. (2019). Constipation-induced pressor effects as triggers for cardiovascular events. J. Clin. Hypertens.

